# Exercise before braces: the mediating effect of pain on the association between physical activity and self-efficacy

**DOI:** 10.3389/fpsyg.2025.1745635

**Published:** 2026-01-12

**Authors:** Caijun Zhao, Zhaohong Sun, Yifan Gong, Tianxiang Cao, Yuan Zhao, Yuan Wen, Zhencheng Li

**Affiliations:** 1 The Affiliated Stomatological Hospital of Chongqing Medical University, Chongqing, China; 2 Chongqing Key Laboratory of Oral Diseases, Chongqing, China; 3 Chongqing Municipal Key Laboratory of Oral Biomedical Engineering of Higher Education, Chongqing, China; 4 Chongqing Municipal Health Commission Key Laboratory of Oral Biomedical Engineering, Chongqing, China; 5 College of Physical Education, Chongqing University, Chongqing, China; 6 Department of Health Sciences, School of Physical Education and Health, Nanchang Institute of Science and Technology, Nanchang, Jiangxi, China

**Keywords:** oral health, orthodontic pain, pain management, physical activity, self-efficacy

## Abstract

**Introduction:**

Self-efficacy is an important psychological factor influencing adherence to orthodontic treatment, and it is itself affected by pain. Although physical activity is associated with pain perception and oral health is closely related to orthodontic pain, the combined effects of these factors on self-efficacy and the mediating mechanism of pain remain unclear.

**Methods:**

295 orthodontic patients were surveyed. Physical activity level was assessed using the Physical Activity Rating Scale-3, oral health with the Oral Health Impact Profile-14, pain with the Numeric Rating Scale, and self-efficacy with the General Self-Efficacy Scale. Mediation analyses were performed with structural equation modeling to test whether pain mediated the relationships between physical activity, oral health, and self-efficacy.

**Results:**

Physical activity was negatively associated with pain (a = −0.018, *p* < 0.001), and pain was negatively associated with self-efficacy (b = −1.124, p < 0.001). The indirect effect of physical activity on self-efficacy through pain was significant (a × b = 0.020, 95% CI [0.009, 0.037]), while the direct effect was not significant, indicating full mediation. Oral health problems were positively associated with pain (a = 0.046, *p* < 0.001) and negatively associated with self-efficacy both directly (c′ = −0.124, *p* = 0.001) and indirectly through pain (a × b = −0.052, 95% CI [−0.092, −0.023]), indicating partial mediation.

**Conclusion:**

Pain serves as a mediator linking preoperative physical activity level and oral health status with self-efficacy. Encouraging patients to engage in regular physical activity and maintain good oral hygiene before orthodontic treatment may strengthen patients’ ability to cope with pain and improve long-term outcomes.

## 1 Introduction

Pain and discomfort are among the most commonly reported adverse effects associated with orthodontic treatment (Oliver and Knapman, 1985; Krishnan, 2007). When orthodontic forces are applied, tooth movement compresses blood vessels in the periodontal tissues and dental pulp, triggering inflammatory responses. On the compressed site inflammatory mediators such as prostaglandins are released, which activate nerve endings in the affected area. These signals are then transmitted via the trigeminal nerve to the brain, resulting in the perception of pain and discomfort (Long et al., 2016; Corrêa et al., 2017). Orthodontic pain typically manifests within 4 h following the placement of arch wires, reaches its peak intensity at approximately 24 h, and subsides almost entirely by the seventh day (Jones, 1984; Kartal, 2016). Orthodontic pain can interfere with patients’ ability to speak and impair sleep quality, and it is particularly aggravated during chewing, prompting changes in dietary habits. Such disruptions can significantly reduce patients’ quality of life (Gao et al., 2021). Moreover, orthodontic pain has the potential to negatively affect patient compliance, including reduced adherence to follow-up appointments and reluctance to use orthodontic appliances. In some cases, severe pain may prompt patients to request slower treatment progression or even to discontinue treatments, thereby jeopardizing the overall treatment outcomes (Roberts et al., 1994; Xie et al., 2023). Given its high prevalence and substantial impact on treatment adherence and patient well-being, the development of effective pain management strategies is of critical clinical importance. Such strategies are essential not only for enhancing the quality of life during orthodontic treatment but also for optimizing therapeutic outcomes.

Over the past several decades, non-steroidal anti-inflammatory drugs (NSAIDs) have remained the most commonly used and effective option for managing orthodontic pain (Teoh et al., 2022; Justi et al., 2025). However, NSAIDs are associated with notable side effects, particularly gastrointestinal damage caused by the inhibition of both cyclooxygenase (COX)-1 and COX-2 enzymes, which reduces the production of gastroprotective prostaglandins (Kotowska-Rodziewicz et al., 2023; Pimenta et al., 2024). Moreover, research has indicated that NSAIDs may slow the rate of tooth movement, potentially compromising treatment outcomes. To address these limitations, COX-2 selective inhibitors were developed as an alternative to conventional NSAIDs. In 1999, the U.S. Food and Drug Administration (FDA) approved rofecoxib, which has a more rapid onset of action and may be useful in treating selected cases of acute postsurgical pain (Moore and Hersh, 2001). However, reports have indicated that rofecoxib is associated with an increased risk of cardiovascular events (Mukherjee et al., 2001; Funk and FitzGerald, 2007; Ahsan and Sahu, 2025), leading to its withdrawal from the market and raising concerns regarding the safety of other COX-2 inhibitors (Stiller and Hjemdahl, 2022). In addition to pharmacological interventions, alternative methods have been introduced for managing orthodontic pain (Li et al., 2024). For instance, the application of the anesthetic gel “Oraqix” and low-intensity infrared laser therapy has demonstrated effectiveness in pain relief (Jamloo et al., 2023; Remi et al., 2023). Mechanical approaches and emerging techniques such as vibration device (Dutta et al., 2025) and gene therapy have also been explored as potential alternatives (Shukla et al., 2023). However, despite their promise, these methods still face significant limitations in terms of practicality, effectiveness, and cost, making them unlikely to replace NSAIDs as a widely used pain management strategy. Therefore, the exploration for safe, effective, and cost-efficient pain management approaches remains a crucial area of research.

Self-efficacy is defined as an individual’s belief in their ability to successfully complete specific tasks (Bandura, 1977). It is widely accepted that individuals with higher self-efficacy are more likely to actively manage postoperative discomfort, adhere to medical recommendations, and maintain optimal oral hygiene practices, all of which contribute to improved orthodontic treatment outcomes (Bandura, 1977; O'leary, 1985). Unsurprisingly, patients’ self-efficacy is negatively correlated with pain perception (Jackson et al., 2014). Postoperative pain not only induces physical discomfort but also exerts a detrimental psychological impact, leading to a reduction in self-efficacy. Consequently, patients with diminished self-efficacy may exhibit reluctance to wear orthodontic appliances or discontinue essential adjunctive treatments, thereby compromising adherence to orthodontic care and potentially undermining treatment success. However, the role of modifiable lifestyle factors, such as prior physical activity and oral health status, in shaping self-efficacy during orthodontic treatment remains unclear.

Pain is a subjective sensation characterized by significant individual variability and influenced by numerous factors, including age, gender, personal pain thresholds, the magnitude of orthodontic force applied, the type of orthodontic appliance, emotional and mental state, cultural background, and prior pain experiences (Apkarian et al., 2005; McGrath, 1994). Among these factors, physical activity is known to influence pain perception through endogenous opioid release, improved circulation, and stress modulation (Sluka et al., 2018). Although physical activity has been established as an effective pain management strategy, postoperative exercise prescriptions are still not routinely employed for pain management in orthodontic treatment. Notably, previous studies have demonstrated an association between prior physical activity level and general pain perception. Specifically, individuals with higher weekly activity levels exhibit lower pain sensitivity to the same stimulus (Am and Ob, 2020; Årnes et al., 2023). Similarly, better oral health reduces inflammation and discomfort, potentially mitigating post-treatment pain (Shang et al., 2023; Kane, 2017). However, whether these factors collectively contribute to enhanced self-efficacy by reducing orthodontic pain has yet to be explored. Understanding this relationship provides a foundation for developing non-pharmacological pre-operative pain management strategies to enhance patient outcomes.

Therefore, this study aims to investigate whether prior physical activity level and oral health status influence self-efficacy by mediating orthodontic pain. We thus propose the following hypotheses:

*H1*: Preoperative physical activity level is positively associated with postoperative self-efficacy.

*H2*: Preoperative oral health status is negatively associated with postoperative self-efficacy.

*H3*: Preoperative physical activity level is negatively associated with postoperative pain.

*H4*: Preoperative oral health status is negatively associated with postoperative pain.

*H5*: Postoperative pain is negatively associated with postoperative self-efficacy.

*H6*: Postoperative pain mediates the relationship between preoperative physical activity level and oral health status with postoperative self-efficacy.

## 2 Methods and materials

### 2.1 Study participants and procedure

The study survey was conducted between January 1, 2024, and April 15, 2025. Participants were interviewed upon their arrival at the Affiliated Stomatological Hospital of Chongqing Medical University for orthodontic visits. The study details were explained to them, and patients who agreed to participated provided written informed consent prior to participation. Four assessment tools were used: the Physical Activity Rating Scale-3 (PARS-3), the Oral Health Impact Profile, 14-item version (OHIP-14), the General Self-Efficacy Scale (GSES), and Numerical Rating Scale-11 (NRS-11) for pain. All questionnaires were administered in paper form, taking approximately 6 minutes to complete across two sessions. The first assessment was conducted prior to orthodontic treatment, during which participants completed the PARS-3 and OHIP-14 questionnaires, analyzed as the pre-treatment data. The second assessment was conducted approximately 24 h after orthodontic treatment—when pain is considered to reach its peak (Ingersoll et al., 1996)—during which participants completed the GSES and the NRS-11 pain assessment.

Participants were included in the study if they were undergoing orthodontic treatment, were at least 18 years of age, and had no prior history of orthodontic treatment. Individuals were excluded if they had clinically confirmed neurological, rheumatological, or psychological disorders (based on psychologist’s reports), had a preexisting chronic orofacial pain condition (either odontogenic or non-odontogenic), were on continuous analgesic, steroid, or neurological therapies, or were unable to read, understand, and/or complete the questionnaire. A total of 564 patients were initially recruited, of whom 295 (58 males and 237 females) completed all assessments. Among the participants, 95 (32.2%) used Ormco Damon™ Q2, 42 (14.2%) used Damon Clear 2, and 158 (53.6%) used Invisalign aligners. Detailed demographic information, including age, height, and weight, is presented in the Results section. The study was conducted in compliance with relevant laws and the ethical guidelines of the Ethics Committee of Chongqing Medical University, and the study was registered under registration number 202404242127000599824.

### 2.2 Measures

#### 2.2.1 PARS-3

The level of physical activity was assessed using the revised version of PARS-3 (Liang, 1994a), which is widely used in the Chinese population (Zou et al., 2024; Tong and Meng, 2023; Yan et al., 2024). This scale evaluates three key components of physical activity: intensity, duration, and frequency, with each component offering five response options scored from 1 to 5. The total physical activity score ranges from 0 to 100 points and is categorized into three levels: low (≤ 19 points), moderate (20–42 points), and high (≥ 43 points). The total physical activity score is calculated using the formula: physical activity score = intensity score × (duration score − 1) × frequency score. The questionnaire has demonstrated good test–retest reliability (*r* = 0.82) (Liang, 1994b) and has been widely used in studies assessing physical activity levels in Chinese populations (Luo et al., 2025; Liu et al., 2025; Wang et al., 2025).

#### 2.2.2 OHIP-14

The OHIP was originally developed by Slade and Spencer (1994) and consisted of 49 items. It was designed to evaluate the negative impacts on functional, psychological, and social well-being stemming from oral health status within a given period. Widely employed in research and clinical practice to assess oral health and hygiene issues, the OHIP was subsequently adapted into a 14-item (Slade, 1997) version and translated into multiple languages (Kenig et al., 2023; Deshpande and Nawathe, 2015; Klotz et al., 2024). Each item in the OHIP-14 questionnaire is assessed using a five-point Likert scale, ranging from 0 (never) to 4 (always). The total score is calculated as the sum of all individual item scores, with a possible range of 0 to 56. Higher scores indicate a greater negative impact on the patient’s quality of life. The Chinese version of the OHIP-14 has demonstrated strong psychometric properties, with excellent internal consistency (Cronbach’s *α* = 0.93) and good construct validity, as indicated by corrected item–total correlations ranging from 0.53 to 0.71, and has been widely applied in Chinese populations with favorable performance (Hongxing et al., 2014; Xin and Ling, 2006; Dongxin et al., 2025).

#### 2.2.3 GSES

GSES was designed by Schwarzer and Jerusalem (1995) to assess individuals’ confidence in successfully accomplishing tasks in various contexts and has been shown to effectively predict motivation, academic and work-related performance, well-being and quality of life. The questionnaire contains 10 items scored on a 4-point Likert scale (1 = “not at all true,” 2 = “hardly true,” 3 = “moderately true,” 4 = “exactly true”). The total score ranges from 10 to 40, with higher scores indicating greater self-efficacy. In this study, we used the Chinese version of the GSES, which has demonstrated satisfactory reliability and validity in previous research (Zhang and Schwarzer, 1995).

#### 2.2.4 NRS-11 for pain

NRS-11 is a unidimensional measure frequently employed to evaluate subjective pain intensity in adults, and it is widely utilized in assessing dental pain. Although multiple versions of the NRS exist, the 11-point scale is most used (Choi et al., 2024; Hartrick et al., 2003). Respondents select an integer from 0 to 10 that best represents their perceived pain intensity at the time of assessment, where “0” denotes “no pain” and “10” denotes “the most severe pain imaginable.” Following the placement of orthodontic appliances, pain typically peaks at approximately 24 h, and therefore, the respondents were surveyed around 24 h post-procedure.

### 2.3 Statistical analysis

Data were analyzed using IBM SPSS Statistics 28.0 for preliminary analyses and AMOS 28.0 for structural equation modeling. First, descriptive statistics were calculated, and independent-samples t-tests were used to examine sex differences in demographic variables, physical activity (PARS-3), oral health (OHIP-14), perceived pain (NRS-11), and self-efficacy (GSES). Pearson’s correlation analysis was then conducted to assess the direction and strength of associations among the main study variables.

To test the hypothesized mediation model, structural equation modeling with maximum likelihood estimation was performed in AMOS. Specifically, the indirect effect of perceived pain on the relationships between oral health, physical activity, and self-efficacy was evaluated. Indirect effects were tested using a non-parametric bootstrap method (5,000 resamples) with bias-corrected 95% confidence intervals (CI). A mediation effect was considered significant when the confidence interval did not include zero. The reliability of the study measures was examined using Cronbach’s α coefficients, all of which indicated good internal consistency. Statistical significance was set at *p* < 0.05 (two-tailed), and results are presented as mean ± standard deviation (SD).

## 3 Results

Table 1 summarizes the demographic and descriptive characteristics of the study sample. A total of 295 participants (58 males and 237 females) were included, with a mean age of 26.39 years (SD = 6.54). The average height, weight, and body mass index (BMI) were 164.44 cm (SD = 7.47), 62.42 kg (SD = 21.90), and 22.98 kg/m^2^ (SD = 7.40), respectively. For the main study variables, the mean scores were 3.55 (SD = 1.51) for pain intensity (NRS-11), 24.87 (SD = 7.14) for general self-efficacy (GSES), 26.40 (SD = 24.16) for physical activity (PARS-3), and 17.81 (SD = 10.46) for oral health-related quality of life (OHIP-14). Independent-samples t-tests indicated significant sex differences in anthropometric measures: males were taller, heavier, and had higher BMI than females (all *p* < 0.05). In contrast, no significant sex differences were observed for age, NRS-11, GSES, PARS-3, or OHIP-14 (all *p* > 0.05). Supplementary Figure 1 presents boxplots of the four primary study variables: pain, GSES, PARS-3, and OHIP-14. The distributions show clear differences in variability across measures. PAIN scores are clustered, indicating relatively low variability in postoperative discomfort. In contrast, PARS-3 displays the widest spread, reflecting substantial individual differences in preoperative physical activity levels. GSES and OHIP-14 demonstrate moderate dispersion, with both variables showing well-defined interquartile ranges and centrally positioned medians.

**Table 1:** Demographic and descriptive characteristics by sex.

Variables	Total (*n* = 295)	Male (*n* = 58)	Female (*n* = 237)	*t*	*p*
Age (year)	26.39 ± 6.54	26.57 ± 6.74	26.34 ± 6.5	0.237	0.813
Height (cm)	164.44 ± 7.47	175.93 ± 5.74	161.63 ± 4.61	20.149	0.000
Weight (kg)	62.42 ± 21.90	77.59 ± 30.47	58.71 ± 17.4	4.541	0.000
BMI	22.98 ± 7.40	24.91 ± 9.18	22.51 ± 6.83	2.227	0.027
NRS-11	3.55 ± 1.51	3.72 ± 1.69	3.5 ± 1.47	1.002	0.317
GSES	24.87 ± 7.14	24.53 ± 7.65	24.95 ± 7.03	−0.400	0.690
PARS-3	26.4 ± 24.16	27.84 ± 26.08	26.05 ± 23.72	0.507	0.612
OHIP-14	17.81 ± 10.46	18.05 ± 11.31	17.75 ± 10.27	0.199	0.843

BMI, body mass index; NRS-11, numerical rating scale-11 for subjective pain level. GSES, general self-efficacy scale; PARS-3, physical activity scale, 3 items; OHIP-14, oral health impact profile, 14 items. *N* = 295.

Pearson’s correlation analysis revealed significant associations among the study variables (Table 2). Pain intensity (NRS-11) was negatively correlated with self-efficacy (GSES; *r* = −0.326, *p* < 0.01) and physical activity level (PARS-3; *r* = −0.314, *p* < 0.01), and positively correlated with oral health problems (OHIP-14; *r* = 0.343, *p* < 0.01). Self-efficacy was positively correlated with physical activity (*r* = 0.171, *p* < 0.01) and negatively correlated with oral health problems (*r* = − 0.269, *p* < 0.01). Physical activity was not significantly correlated with oral health problems (*r* = −0.074, ns).

**Table 2:** Pearson’s correlation analysis of each variable.

Variables	NRS-11	GSES	PARS-3	OHIP-14
NRS-11	1.0			
GSES	−0.326**	1.0		
PARS-3	−0.314**	0.171**	1.0	
OHIP-14	0.343**	−0.269**	−0.074	1.0

NRS-11, numerical rating scale-11 for subjective pain level. GSES, general self-efficacy scale; PARS-3, physical activity scale, 3 items; OHIP-14, oral health impact profile, 14 items. ** indicates *p* < 0.01. *N* = 295.

The hypothesized mediation model demonstrated good fit to the data, χ^2^(1) = 1.63, *p* = 0.202, CFI = 0.994, TLI = 0.964, GFI = 0.997, RMSEA = 0.046 (90% CI [0.000, 0.170], PCLOSE = 0.354), SRMR = 0.006. The predictors explained 19% of the variance in pain (R^2^ = 0.19) and 14% of the variance in self-efficacy (R^2^ = 0.14), suggesting that prior physical activity and oral health status accounted for a meaningful portion of variability in pain and, through pain, contributed modestly to explaining self-efficacy.

**Figure 1:**
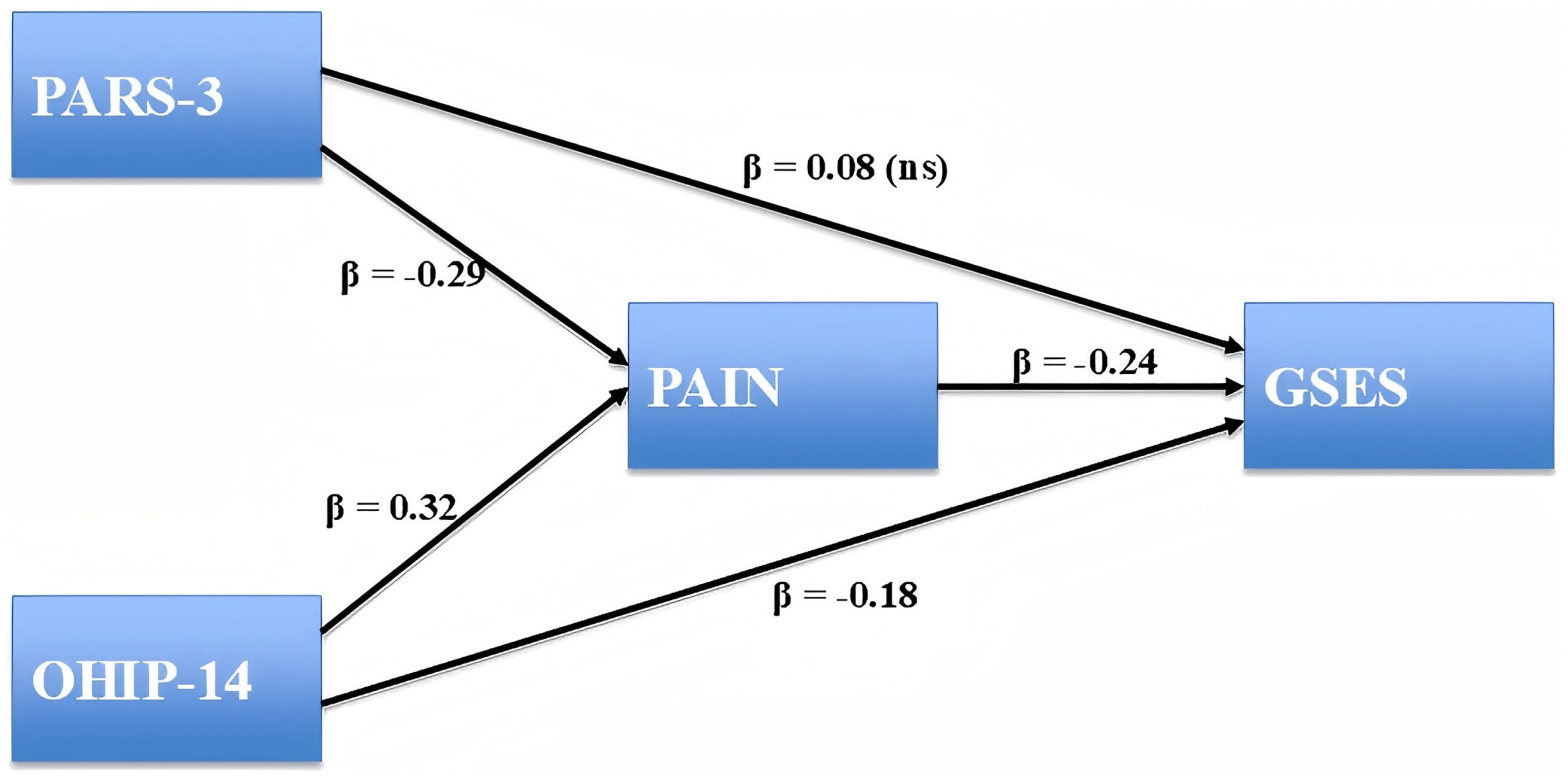
Structural equation model testing the mediating role of pain. Standardized path coefficients (*β*) are displayed. Nonsignificant paths are marked as “ns.” Physical Activity Rating Scale-3 (PARS-3), Oral Health Impact Profile-14 (OHIP-14), and General Self-Efficacy Scale (GSES). *N* = 295.

As shown in Table 3, physical activity (PARS3) significantly predicted lower pain (a = −0.018, SE = 0.003, *p* < 0.001), and pain in turn significantly predicted lower general self-efficacy (GSES; b = −1.124, SE = 0.286, *p* < 0.001). The direct effect of physical activity on self-efficacy was not significant (c′ = 0.025, SE = 0.017, *p* = 0.143), while the indirect effect through pain was significant (a × b = 0.020, 95% CI [0.009, 0.037], *p* < 0.01). The total effect was also significant (c = 0.045, SE = 0.017, *p* < 0.01), indicating that the relationship between physical activity and self-efficacy was fully mediated by pain. Similarly, oral health problems (OHIP-14) were positively associated with pain (a = 0.046, SE = 0.008, *p* < 0.001), and pain was negatively associated with self-efficacy (b = −1.124, SE = 0.286, *p* < 0.001). The direct effect of oral health on self-efficacy remained significant (c′ = −0.124, SE = 0.039, *p* = 0.001), and the indirect effect via pain was also significant (a × b = −0.052, 95% CI [−0.092, −0.023], *p* < 0.01). The total effect of oral health on self-efficacy was significant (c = −0.176, SE = 0.036, *p* < 0.001), consistent with partial mediation by pain. The standardized path coefficients for the mediation model are presented in Figure 1. Higher level of physical activity was indirectly associated with higher self-efficacy through reduced pain, while oral health problems exerted both direct and indirect negative effects on self-efficacy.

**Table 3:** Mediation analysis of physical activity (PARS-3) and oral health (OHIP-14) on self-efficacy (GSES) through pain (NRS-11).

Model	Variable			Effect of X on M	Effect of M on Y	Direct effect	Indirect effect	Total effect
	X (Predictor)	M (Mediator)	Y (Outcome)	a	b	c′	(a*b) [95% CI]	c = c′ + a*b
1	PARS	Pain	GSES	−0.018 (SE = 0.003)***	−1.124 (SE = 0.286)***	0.025 (SE = 0.017), ns	0.020 [0.009, 0.037]**	0.045 (SE = 0.017)**
2	OHIP14			0.046 (SE = 0.008)***		−0.124 (SE = 0.039)**	−0.052 [−0.092, −0.023]**	−0.176 (SE = 0.036)***

SE, standard error; PARS-3, physical activity rating scale, 3 items for physical activity level; OHIP-14, oral health impact profile, 14 items, for oral health-related quality of life. Indirect effect is significant if 0 value falls outside the lower bound and upper bounds of bootstrap CI. **p < 0.01, ***p < 0.001, ns, non-significant. *N* = 295.

## 4 Discussion

The current study underscores the mediating role of subjective pain in linking preoperative physical activity level and oral health with postoperative self-efficacy among orthodontic patients. The results indicate that physical activity was indirectly associated with greater self-efficacy through its effect on pain, with no significant direct effect. By contrast, oral health influenced self-efficacy both directly and indirectly through pain. Specifically, better oral health status before orthodontic treatment was associated with lower perceived pain, which in turn contributed to greater self-efficacy. These findings align with prior research suggesting that individuals with higher levels of physical activity have lower pain sensitivity to the same stimulus (Årnes et al., 2023), reinforcing the role of exercise in pain management (Am and Ob, 2020; Årnes et al., 2023). Similarly, our results support the findings of Hadler-Olsen et al. (2024) who reported that poor periodontal health exacerbates pain perception through inflammation, whereas good oral hygiene mitigates discomfort. Additionally, the observed negative correlation between pain and self-efficacy supports psychological models suggesting that self-efficacy is influenced by negative subjective sensation (Cheng et al., 2018; Kardash et al., 2024). While previous studies have independently examined the effects of physical activity and oral health on pain perception, the present study makes a novel contribution by identifying pain as a mediating factor between these variables and self-efficacy. Specifically, the results showed that the effect of physical activity on self-efficacy was fully mediated by pain, whereas the effect of oral health was partially mediated by pain, with both direct and indirect pathways. These findings offer a more comprehensive understanding of the interrelationships among physical, behavioral, and psychological factors in orthodontic treatment outcomes.

From a clinical perspective, these findings support the incorporation of recommendations for regular physical activity and improved oral hygiene into pre-treatment orthodontic care as non-pharmacological strategies to enhance patient outcomes. Given that orthodontic treatment typically lasts 18–24 months, maintaining an active lifestyle and good oral hygiene throughout the treatment process may further strengthen patients’ self-efficacy in coping with discomfort, thereby contributing to better long-term results (Naseri et al., 2020; Szczepańska-Gieracha and Mazurek, 2020). Regular physical activity is known to increase pain tolerance through multiple physiological mechanisms, including the release of endogenous opioids, improved circulation, faster resolution of inflammation, and regulation of stress-related hormones such as cortisol (Am and Ob, 2020). Similarly, maintaining good oral hygiene reduces the accumulation of dental plaque and gingival inflammation, decreases periodontal perception, and lowers the risk of secondary infections, all of which can alleviate treatment-related discomfort (Luchian et al., 2024). Collectively, these findings highlight the importance of integrating lifestyle-based interventions into orthodontic pain management to optimize patient well-being.

This study has several limitations that should be acknowledged when interpreting the findings. First, its cross-sectional design prevents the establishment of causal relationships; future research should adopt longitudinal or interventional approaches to further verify the mediating role of pain between physical activity, oral health, and self-efficacy. Second, the levels of physical activity, oral health status, and pain were assessed using self-reported questionnaires, which may be subject to recall and social desirability biases. Objective measures such as accelerometer-based activity monitoring, clinical periodontal examinations, and biomarkers of pain should be incorporated in future studies to improve measurement accuracy. Third, psychosocial factors such as anxiety, stress, and sleep quality, which may also influence pain perception and self-efficacy, were not included in the current analysis (Sobol-Kwapinska et al., 2016). Future research should integrate these variables to provide a more comprehensive understanding of the underlying mechanisms.

## 5 Conclusion

These findings highlight the critical role of pre-treatment physical activity and oral health in enhancing self-efficacy among orthodontic patients through the reduction of pain. The results suggest that incorporating non-pharmacological strategies—such as promoting regular physical activity and maintaining optimal oral hygiene—into orthodontic care before treatment may strengthen patients’ ability to cope with discomfort, thereby improving treatment adherence and long-term outcomes.

## Data Availability

The raw data supporting the conclusions of this article will be made available by the authors, without undue reservation.

## Supplementary material

SUPPLEMENTARY FIGURE 1 Boxplots of the primary study variables. This figure displays the distribution of postoperative pain (PAIN), self-efficacy (GSES), physical activity level (PARS-3), and oral health impact (OHIP-14). The boxplots present medians, interquartile ranges, and overall score dispersion, offering a visual summary of the variability across the key study measures.
