# Supplementary figures and images for: Exercise before braces: the mediating effect of pain on the association between physical activity and self-efficacy
Source: Front Psychol. 2026 Jan 12;16:1745635. doi: 10.3389/fpsyg.2025.1745635 (PMC12832512; doi:10.3389/fpsyg.2025.1745635)

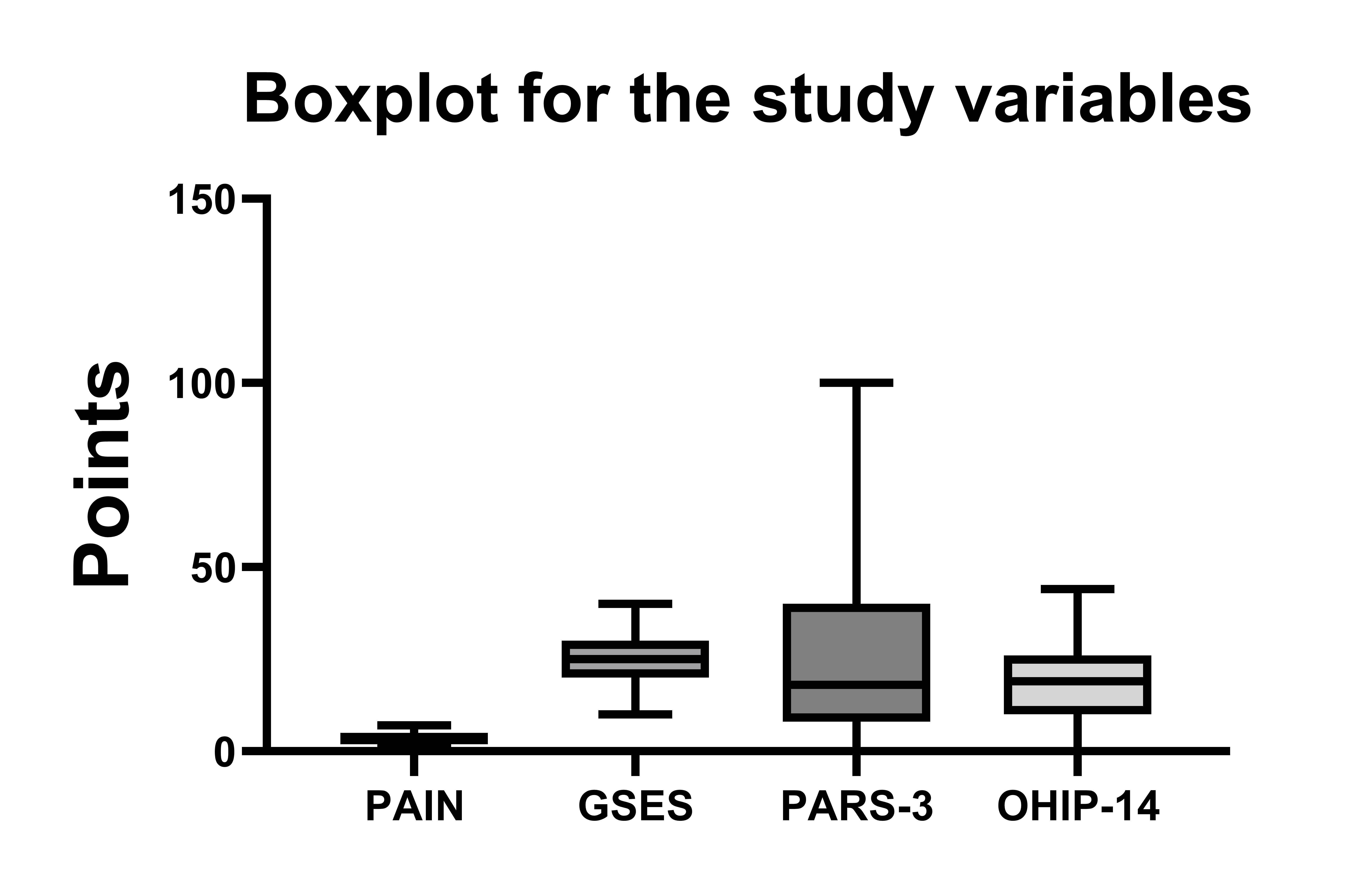

Supplement: SUPPLEMENTARY FIGURE 1 — Boxplots of the primary study variables. This figure displays the distribution of postoperative pain (PAIN), self-efficacy (GSES), physical activity level (PARS-3), and oral health impact (OHIP-14). The boxplots present medians, interquartile ranges, and overall score dispersion, offering a visual summary of the variability across the key study measures. [file Image_1.TIF]
